# NGS Data Repurposing Allows Detection of tRNA Fragments as Gastric Cancer Biomarkers in Patient-Derived Extracellular Vesicles

**DOI:** 10.3390/ijms24108961

**Published:** 2023-05-18

**Authors:** Joaquín J. Maqueda, Mafalda Santos, Marta Ferreira, Sérgio Marinho, Sara Rocha, Mafalda Rocha, Nadine Saraiva, Nuno Bonito, Joana Carvalho, Carla Oliveira

**Affiliations:** 1 i3S—Instituto de Investigação e Inovação em Saúde, Universidade do Porto, 4200-135 Porto, Portugalmafalda.santos@medicina.ulisboa.pt (M.S.);; 2 Bioinf2Bio LDA, 4200-150 Porto, Portugal; 3 IPATIMUP—Instituto de Patologia e Imunologia Molecular da Universidade do Porto, 4200-135 Porto, Portugal; 4 Department of Medical Sciences, Institute of Biomedicine—iBiMED, University of Aveiro, 3810-193 Aveiro, Portugal; 5 Instituto Português de Oncologia de Coimbra Francisco Gentil, E.P.E. (IPOCFG, E.P.E.), 3000-075 Coimbra, Portugal; 6 Department of Pathology, Faculty of Medicine, University of Porto, 4200-319 Porto, Portugal

**Keywords:** tRNA fragments, extracellular vesicles, gastric cancer, NGS data repurposing, cancer biomarkers

## Abstract

Transfer RNA fragments (tRFs) have gene silencing effects similarly to miRNAs, can be sorted into extracellular vesicles (EVs) and are emerging as potential circulating biomarkers for cancer diagnoses. We aimed at analyzing the expression of tRFs in gastric cancer (GC) and understanding their potential as biomarkers. We explored miRNA datasets from gastric tumors and normal adjacent tissues (NATs) from TCGA repository, as well as proprietary 3D-cultured GC cell lines and corresponding EVs, in order to identify differentially represented tRFs using MINTmap and R/Bioconductor packages. Selected tRFs were validated in patient-derived EVs. We found 613 Differentially Expressed (DE)-tRFs in the TCGA dataset, of which 19 were concomitantly upregulated in TCGA gastric tumors and present in 3D cells and EVs, but barely expressed in NATs. Moreover, 20 tRFs were expressed in 3D cells and EVs and downregulated in TCGA gastric tumors. Of these 39 DE-tRFs, 9 tRFs were also detected in patient-derived EVs. Interestingly, the targets of these 9 tRFs affect neutrophil activation and degranulation, cadherin binding, focal adhesion and the cell–substrate junction, highlighting these pathways as major targets of EV-mediated crosstalk with the tumor microenvironment. Furthermore, as they are present in four distinct GC datasets and can be detected even in low quality patient-derived EV samples, they hold promise as GC biomarkers. By repurposing already available NGS data, we could identify and cross-validate a set of tRFs holding potential as GC diagnosis biomarkers.

## 1. Introduction

Over 1 million people were diagnosed with Gastric Cancer (GC) in 2020, making it the fifth most commonly diagnosed type of cancer [1]. GC ranks fourth in mortality rates due to a diagnosis at a late disease stage, where the median overall survival (OS) ranges from 4.6 to 13.1 months [1,2]. A GC diagnosis is usually invasive, resorting to endoscopy with biopsy, as well as staging with endoscopic ultrasound and CT scans [3]. Therefore, there is an increasing interest in less invasive molecular biology-based approaches both for a GC diagnosis and prognosis [4].

The detection of GC biomarkers in body fluids, such as blood or urine, is already in testing, with some studies showing promising results [5]. For instance, the detection of miR-21 in serum or in human peripheral blood mononuclear cells (PBMCs) is claimed to predict GC in 90% of the studied cases [6]. In another study, a panel of six upregulated miRNAs was found both in serum and GC Extracellular Vesicles (GC-EVs), showing that GC-EV cargo can be a useful diagnosis marker [7]. EVs are essential intercellular communication mediators, carrying mostly non-coding RNAs (ncRNAs), mRNAs, DNAs, lipids and proteins [8]. This cargo is highly affected by cellular architecture and culture conditions, with EVs deriving from 3D-cultured cancer cells recapitulating better EVs recovered from real cancer patients [9,10]. Studies using EV-ncRNAs are usually focused on miRNAs; however, some studies report that up to 50% of their cargo is actually transfer RNAs (tRNAs) and tRNA-derived fragments (tRFs) [9,11,12,13,14].

The interest in the role of tRNAs in cancer has risen in the past few years and, since then, tRNA deregulation has been associated with tumor initiation, tumor growth, metastasis and bad prognosis [15,16,17,18] (reviewed in [19]). There are two main species of non-coding RNAs derived from tRNAs: tRFs and tRNA halves (tiRNAs) [20]. While tiRNAs result from cleavage at the anticodon loop under stress [21,22], tRFs are 14–30 nucleotide fragments that derive from mature or pre-tRNAs and can control gene expression similarly to miRNAs [23]. tRFs can be further subdivided into 3′-tRFs or 5′-tRFs if they include the terminal 3′ or 5′ portion of the original tRNA, or i-tRFs if they originate from an internal tRNA sequence. Several tRFs have been implicated in many diseases, from cancer to neurodegenerative and metabolic disorders [24,25,26]. Moreover, tRFs sorted into EVs are being evaluated for their value as non-invasive cancer diagnostic biomarkers [27,28]. A recent study has identified eight differentially expressed tRFs between GC tissues and adjacent tissues [29], with tRF-24-V29K9UV3IU regulating the Wnt pathway to inhibit cell proliferation, migration and invasion, while promoting cell apoptosis. Additionally, a downregulation of tRF-Glu-TTC-027 was observed in GC patients, which acts as a tumor suppressor, inhibiting the MAPK pathway and negatively influencing tumor progression [30]. On the other hand, tRF-3017A was shown to silence the tumor suppressor NELL2, promoting cell migration and invasion in vitro, and it is associated with a higher lymph node metastasis in GC patients [31]. Moreover, expression levels of hsa_tsr016141 in serum could distinguish GC patients from healthy donors and gastritis patients, with good sensitivity, specificity and holding potential to be used for the dynamic monitoring of GC patients [32].

Due to the mandatory deposition of sequencing data in online repositories, there are numerous accessible datasets nowadays. These datasets can be explored to retrieve different information from what is depicted in corresponding initial publications. This is an interesting savings strategy, avoiding unnecessary experiments and costs. An example might be the use of small-RNAseq datasets used to evaluate miRNA deregulation, but containing information about other small RNAs such as tRFs, due to their similar RNA length. In this manuscript, we used miRNA datasets from the TCGA database to evaluate tRF modulation in GC patients. The deregulated tRFs were validated with another small-RNAseq dataset from our own laboratory, previously used to assess miRNA deregulation. We found tRFs with positive and negative correlations in TCGA tumors and normal counterparts, as well as in EVs produced by 3D-cultured GC-cell lines. Nine tRFs recurrently identified in the previous analysis were also detected in GC patient plasma-derived EVs. Besides their biomarker potential, these differentially expressed (DE)-tRFs were predicted to modulate the tumor microenvironment and affect cell–cell adhesion.

## 2. Results

### 2.1. tRFs Are Highly Expressed in GC Tumors

**Figure 1:**
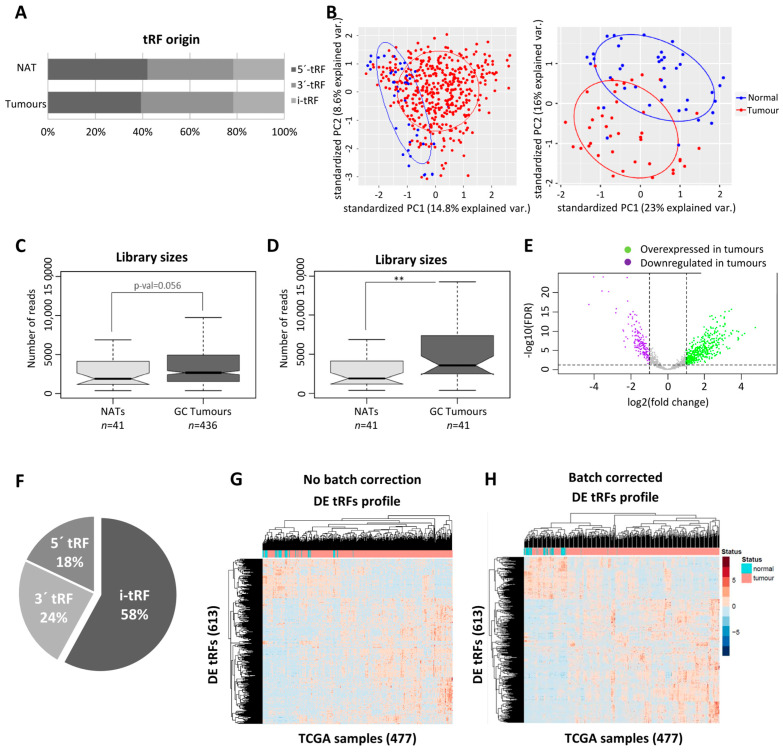
tRFs are mostly upregulated in TCGA GC tumors. (**A**) tRF biotype distribution is similar in GC tumors and NAT. (**B**) PCA analysis showing tumor and NAT distinction according to tRF expression in all TCGA GC datasets (**left panel**) and in paired samples (**right panel**). Ellipse confidence interval = 68%. (**C**) tRFs have a tendency to be more abundant in GC tumors than in NAT (full TCGA dataset). (**D**) tRFs are more abundant in GC tumors than in NAT (paired samples) (** *p* < 0.01). (**E**) Volcano plot showing the distribution of differentially expressed (DE)-tRFs. In GC tumors, 613 tRFs were differentially expressed, from which 446 were upregulated and 167 were downregulated in tumors vs. NATs (|log_2_FC| > 1 and an *adj.p-val* < 0.05). (**F**) Pie chart showing the biotype distribution of the DE-tRFs. (**G**) Heatmap before batch correction showing the differential expression of tRFs in each GC tumor and NAT sample analyzed in the TCGA dataset. (**H**) Heatmap after batch correction, showing a better discrimination of GC tumors and NAT based on DE-tRF profiles.

To have a broad perception of the expression of tRFs in GC, we analyzed the miRNAseq data of stomach adenocarcinomas (*n* = 436) and normal adjacent tissues (*n* = 41) deposited in the TCGA database. Our first finding was that the TCGA miRNAseq dataset is devoid of tRNA halves and enriched in 5′ and 3′ tRFs. The biotype distribution of tRFs is very similar between tumors and normal adjacent tissues (NATs) (Figure 1A). The PCA analysis, comparing tRF expression in all tumors against all NATs, hinted to a separation of tumors from NATs (Figure 1B, left panel). This separation became more evident when tumors and normal adjacent tissue coming from the same patient (paired NAT samples) were analyzed (Figure 1B, right panel). To increase confidence in resulting data, a tRF expression threshold of 5 Reads per Million (RPMs) in at least 20% of samples from a given condition (tumor or NAT) was used. After application of this filter, we analyzed the total tRF reads and observed that tumors have an overall increase in tRF expression, especially if paired samples are considered (*p* < 0.01) (Figure 1C,D).

The differential expression (DE) analysis of tRFs considering |log_2_(fold-change)| > 1 and an adjusted *p*-value of <0.05 unveiled 613 DE-tRFs, from which 446 were upregulated and 167 were downregulated in tumors vs. NATs (Figure 1E). While 5′- and 3′-tRFs were the most abundant species among tRFs from tumors and NATs (Figure 1A), the most abundant biotype among DE-tRFs was i-tRFs (58%), followed by 3′-tRFs (24%) and 5′-tRFs (18%) (Figure 1F). These 613 DE-tRFs, when plotted into a heatmap, unveiled a batch effect that has been previously described by other TCGA users [33] (Figure 1G). After batch effect correction, gastric tumors and NAT samples clustered quite distinctly based on the 613 tRFs’ expression (Figure 1H). The top fifteen DE-tRFs encompassed eleven downregulated tRFs (nine i-tRFs and two 3′-tRFs) and four upregulated tRFs (one i-tRF and three 3′-tRFs) (Table 1).

### 2.2. tRF Expression in GC Cell Lines and Derived EVs

To assess a potential role for tRFs as biomarkers, we next assessed their expression in a small-RNAseq dataset from EVs produced by two different 3D-cultured GC cell lines (MKN74 and MKN45). We chose to focus our analysis on GC cell lines and EVs grown in 3D cultures, as they resemble more of the context of tumors growing in patients and often their expression patterns are closer to the in vivo context. We detected a distinct landscape of tRF species from what we observed in TCGA, possibly because these datasets were sequenced on different platforms (TCGA—Illumina; in-house datasets—Ion Torrent). Besides this difference, the TCGA method only allows for the detection of fragments below 30 bp, as this was the read length chosen for sequencing. tRNA halves are usually longer than 30 bp, therefore they are not represented in the TCGA dataset. The Ion Torrent sequencing method used in our datasets had a higher read length (<100 bp), so we could evaluate the presence of tRNA halves.

**Figure 2:**
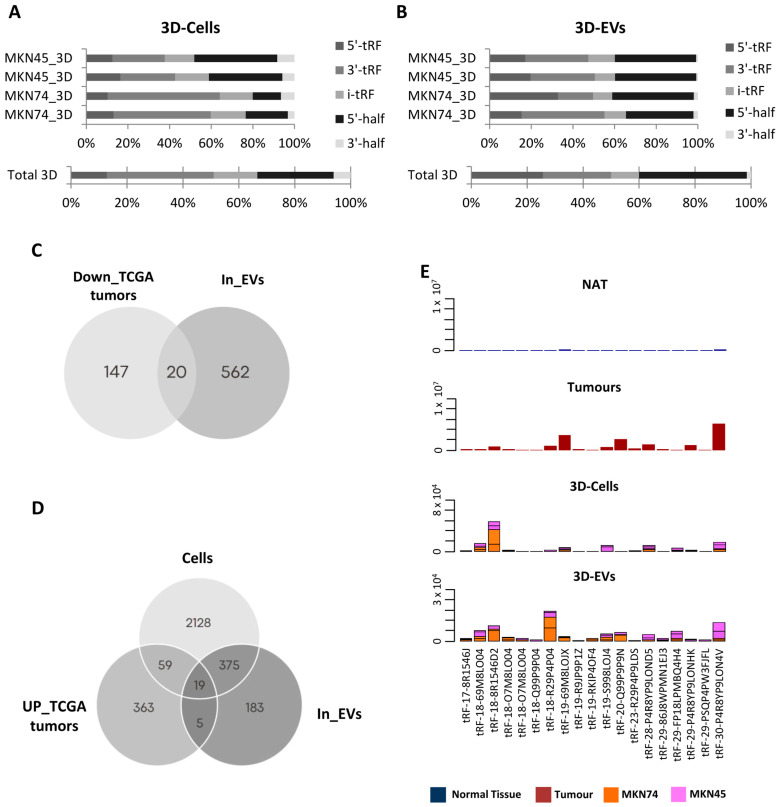
tRF characterization in 3D-GC cell lines and respective EVs. (**A**) tRF biotype distribution in MKN45 and MKN74 grown in 3D shows overall enrichment of 3′-tRFs. (**B**) tRF biotype distribution in EVs derived from MKN45 and MKN74 grown in 3D shows overall enrichment of 5′-halves. (**C**) Venn diagram showing that from the 167 tRFs downregulated in TCGA tumors vs. NAT, 20 are also present in EVs derived from 3D-GC cell lines. (**D**) Venn diagram showing that from the 447 tRFs upregulated in TCGA tumors vs. NAT, 19 are also present in 3D-GC cell lines and their derived EVs. (**E**) Graphics depict the number of Reads per Million (RPMs) of the 19 tRFs found to be upregulated in TCGA GC tumors vs. NAT and present in 3D-GC cell lines and their derived EVs in NAT, tumors, 3D-grown GC cell lines and their derived EVs.

In 3D cells, the most abundant tRF species was 3′-tRFs, followed by 5′-halves, i-tRFs, 5′-tRFs and 3′-halves (Figure 2A). In cell line-derived EVs, 5′-halves were the most abundant species, followed by 5′-tRFs, 3′-tRFs, i-tRFs and 3′-halves (Figure 2B). These differences may reflect an asymmetric and orchestrated tRF packaging into EVs, in order to modulate the surrounding microenvironment, as it has been reported in T-cells [34].

As RNA packaging into EVs does not seem to be random, we aimed at identifying tRFs that can be clinically useful to diagnose or stratify GC patients. For that, we used two strategies to find potentially relevant tRFs. First, we searched EVs from 3D-GC cells for the 167 downregulated tRFs in TCGA tumors compared to NATs, assuming the hypothesis of tRF active exclusion by tumor cells. In a second approach, we searched in 3D-GC cells and their respective EVs for the 446 upregulated tRFs in TCGA tumors compared to NATs, assuming the hypothesis of an active load of tRFs in cancer-derived EVs.

We found 20 tRFs in 3D-GC EVs for which expression has a negative correlation between NATs and TCGA tumors, being hypothetically packed into EVs to avoid a potential negative impact on the tumor cell fitness (Figure 2C). Additionally, we found 19 tRFs that were concomitantly upregulated in TCGA tumors and present in 3D cells and respective EVs (Figure 2D). These tRFs were barely expressed in normal adjacent tissue from TCGA (NATs) and for most cases their expression levels have similar levels to those in 3D-GC cells and their derived EVs (Figure 2E). Interestingly, no correlation was found between the expression levels of tRFs in 3D cells and respective EVs (Supplementary Table S1).

### 2.3. DE-tRFs Are Predicted to Modulate Immune Response and Cell Adhesion

**Figure 3:**
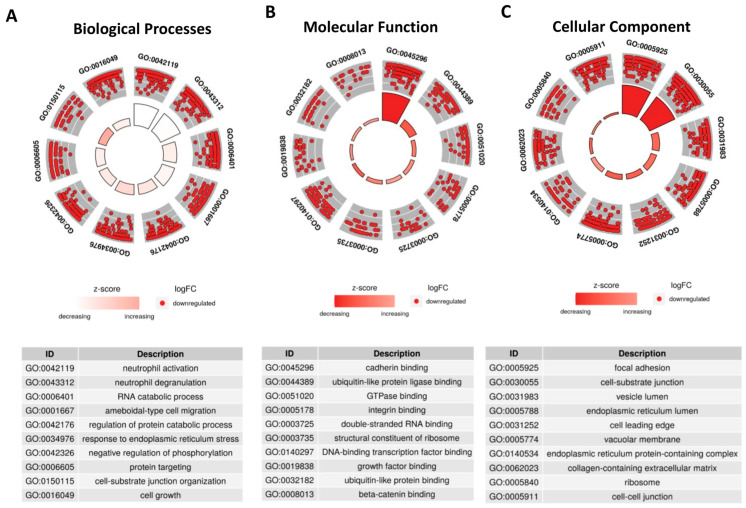
DE-tRFs can modulate immune response and cell adhesion. Using tRFTar, the targets of the 39 DE-tRFs were predicted and GO term analysis was performed using ClusterProfiler R package. The GO terms predicted to be affected by these tRFs are depicted in circular plots. (**A**) The top two categories in biological processes predicted to be deregulated are related to neutrophil activation and degranulation, showing these tRFs can impact local immune response. (**B**) The molecular function predicted to be more impacted by these tRFs was cadherin binding. (**C**) Focal adhesion and the cell-substrate junction are the top deregulated categories in cellular component GO terms.

Since these 39 DE-tRFs have all been found within EVs from 3D-GC cells, we proceeded to predict their targets in recipient cells using the tRFTar tool. The predicted targets were then used to perform a GO term analysis using the *ClusterProfiler* R package to assess their impact in cellular biology (Figure 3).

Regarding biological processes, DE-tRFs were found to affect neutrophils and their degranulation (Figure 3A). Interestingly, these tRFs also seem to affect cell adhesion properties, as they target genes that are involved in ameboidal-type cell migration (Figure 3A) and cadherin, β-catenin and integrin binding (Figure 3B), among other adhesion-related categories, such as focal adhesion and the cell–substrate junction (Figure 3C). We also separately analyzed the GO terms affected by the targets of up- and downregulated tRFs. The results are very similar in both datasets since these tRF target genes have similar functions and their interaction network is redundant (Supplementary Figure S1).

Therefore, besides their potential role as biomarkers for GC diagnoses, these DE-tRFs may also affect central pathways involved in gastric carcinogenesis and progression.

### 2.4. Nine DE-tRFs Are Also Present in Patient-Derived EVs

**Figure 4:**
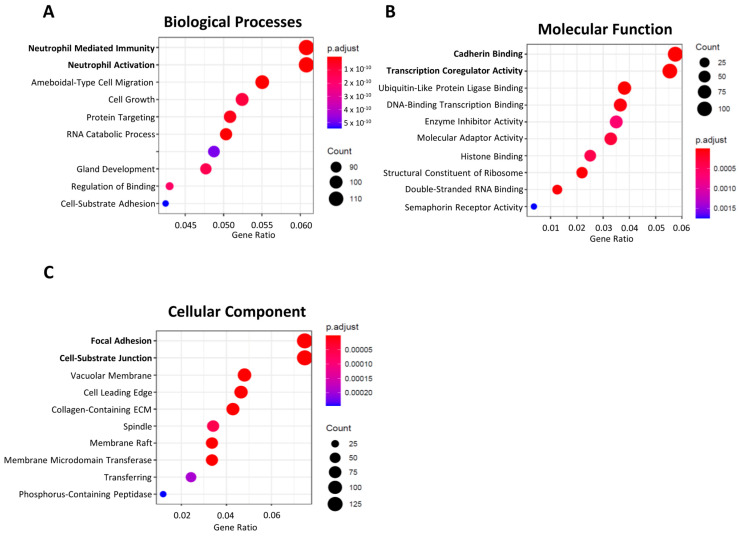
The nine DE-tRFs present in patient-derived EVs are also predicted to impact immune response and cell adhesion. (**A**–**C**) Dotplots showing GO term groups in Biological Processes, Molecular Function and Cellular Component, predicted to be impacted by the 9 DE-tRFs present across all datasets. The top GO terms are similar to what is observed in Figure 3.

We explored whether any of the 39 tRFs, which were found to be differentially expressed in TCGA tumors vs. NAT and present in GC-EVs, could be found in a small-RNAseq dataset from plasma EVs obtained from four GC patients, prior to their GC surgery. Despite the low number of patients and low input material, we could validate the presence of nine DE-tRFs in patient-derived EVs. Six of these DE-tRFs were downregulated in TCGA tumors vs. NAT, but present in EVs from 3D-GC cells; three DE-tRFs were upregulated in TCGA tumors vs. NAT and present in both 3D-GC cells and respective 3D-EVs. The tRF ID, sequence and type are described in Table 2. This validates our strategy to find relevant biomarkers from previously available data. We predicted the targets of these 9 tRFs using the same strategy we used to predict the targets of the 39 tRFs. These targets were then used to perform a GO term analysis. Interestingly, the targets of these nine tRFs also affect neutrophil activation and degranulation, cadherin binding, focal adhesion and the cell-substrate junction, highlighting these pathways as probable targets of EV-mediated crosstalk with the tumor microenvironment (TME) (Figure 4A–C).

**Figure 5:**
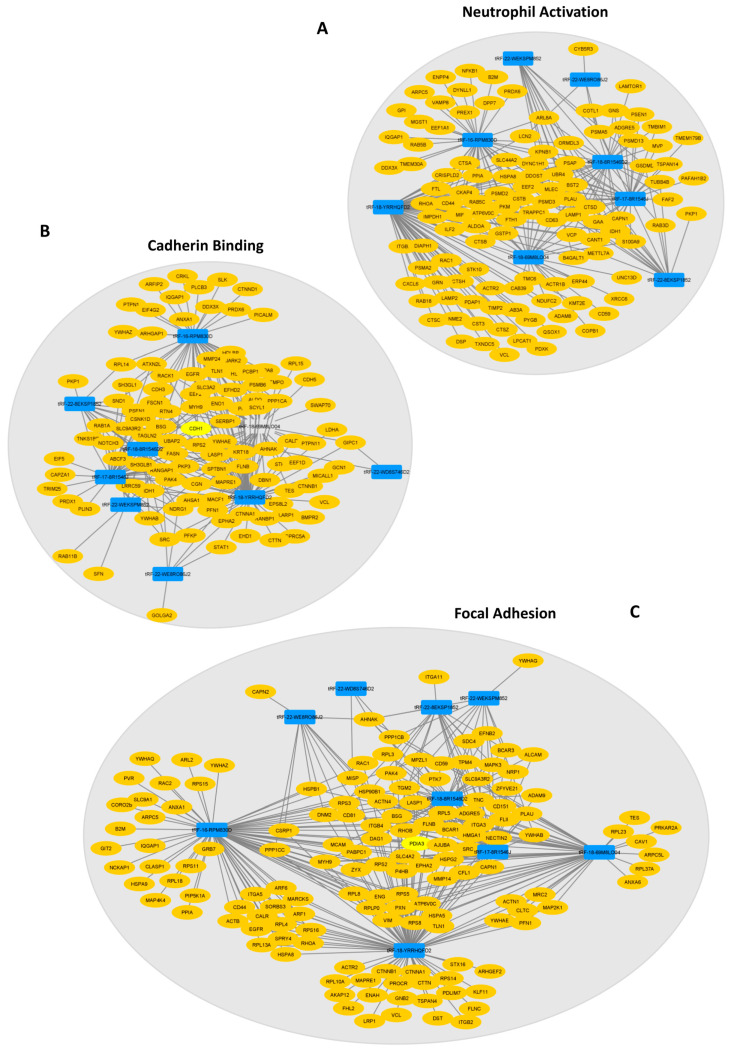
Interaction networks show how tRFs are predicted to impact the top GO terms of each category. (**A**) “Neutrophil Activation” network depicting 8 tRFs interacting with their predicted targets. (**B**) “Cadherin Binding” interaction network showing the links between 8 tRFs and the predicted targets, having *CDH1* as a central node. (**C**) All nine DE-tRFs present in patient EVs are predicted to impact the expression of genes that influence “Focal Adhesion” (central network node: *PDIA3*). Legend: Blue rectangles: tRFs; yellow ovals: predicted targets.

To understand how DE-tRFs and their targets are interacting to affect these biological processes, we generated interaction networks in Cytoscape and observed that almost all nine DE-tRFs can target genes that affect the top GO terms in each group (Figure 5). These interaction networks are very complex, with each DE-tRF being able to target more than one gene, and several genes having seed sequences where distinct DE-tRFs can bind (Figure 5). The top biological process affected is neutrophil activation (GO:0042119) with eight DE-tRFs being predicted to affect 110 genes in this process (Figure 5A). Cadherin binding (GO:0045296) is the most affected molecular function, with all the DE-tRFs affecting 100 genes. Interestingly, *CDH1*, the gene coding for E-cadherin, is a central node of this network, being predicted to be targeted by five out of nine DE-tRFs (Figure 5B). It is also remarkable that one of the central nodes of the “Focal Adhesion” network (GO:0005925) is *PDIA3*, a molecular chaperone reported to influence antigen presentation in gastric cancer, and thus likely to influence the therapeutic response (Figure 5C). In fact, low levels of this protein often correlate with immune evasion and the worst overall survival [35].

In summary, we identified nine DE-tRFs across five different GC-related datasets, including patient-derived EVs, and with neglectable expression in normal gastric tissue, that may work both as diagnosis biomarkers and as modulators of GC behavior and the related microenvironment.

## 3. Discussion

Our work demonstrated that it is possible to draw a new and relevant hypothesis with the sole use of an in silico de novo data analysis. Using data available in public repositories and previously sequenced data in our lab, we could confirm that nine DE-tRFs with a negative or positive correlation between tumors and GC cell line-derived EVs are also present in patient-derived EVs (Figure 4A).

We started by using TCGA data to assess DE-tRFs between tumors and normal adjacent tissues. This analysis unveiled that GC tumors express more tRFs than NAT (Figure 1C,D), in agreement with previous reports [36]. Interestingly, a report showed that angiogenin, one of the enzymes responsible for cleaving tRNAs into tRFs, is more active in GC tumors than in NAT [37]. In fact, we observed most DE-tRFs were overexpressed in tumors (Figure 1E), likely reflecting greater angiogenin activity. When plotting the DE-tRFs into a heatmap, we noticed a batch effect in our samples related to the center where the samples were sequenced (Figure 1G), as previously reported [33]. Therefore, we included cancer status and batch in our linear model prior to the differential expression analysis to exclude bias. After bias exclusion, we observed that the 613 DE-tRFs could discriminate GC samples from normal tissue fairly well (Figure 1H).

As we were set to find tRFs with biomarker values, we validated the 613 DE-tRFs from TCGA with small-RNAseq data from two different GC cell lines cultured in 3D and their respective EVs. Patient EVs can be obtained from liquid biopsies, an increasingly popular non-invasive method to monitor patients and people with an increased risk to develop disease [38]. We decided to use 3D-GC cell lines as a proxy, since EVs derived from 3D cell cultures better resemble the content of patient-derived EVs than 2D cultures [9,39,40]. We found 20 tRFs that were downregulated in TCGA tumors but present in EVs from 3D-GC cells, and 19 tRFs that were upregulated in TCGA tumors and present in both 3D-GC cells and in corresponding EVs (Figure 2C,D). RNA packaging into EVs is not completely random, depending on cell type and physiological conditions [41]. Although the mechanisms for active RNA loading into EVs still need clarification, EXOmotifs were described in miRNAs that are enriched in neuron-derived EVs [42]. Nevertheless, it is still controversial as to which proportion of EV content is selected for and which portion of their content is sampled by the nature of the EV biogenesis process [43]. In addition, the annotation and databases of tRFs still need to be improved. tRF reads can sometimes map to several distinct tRFs, since they have very similar (or identical) sequences, which make it difficult to differentiate between them. Interestingly, the tRFs that we found to have a positive or negative expression correlation between GC-EVs and TCGA tumors seem to have the potential to modulate the tumor microenvironment (Figure 3). The crosstalk between tumor cells and the stromal cells in the TME mediated by EVs produces important functional changes that favor tumor development and progression [44,45,46]. Indeed, we observed that the targets affected by these tRFs interfere with neutrophil activation, cell–substrate adhesion, focal adhesion, extracellular matrix organization and cadherin binding (Figure 3), which are relevant in gastric cancer biology [47,48,49,50,51]. Even if we consider only the nine tRFs, which are also present in patient EVs, their targets modulate the exact same pathways (Figure 4A–C), suggesting a highly selected biological process.

**Figure 6:**
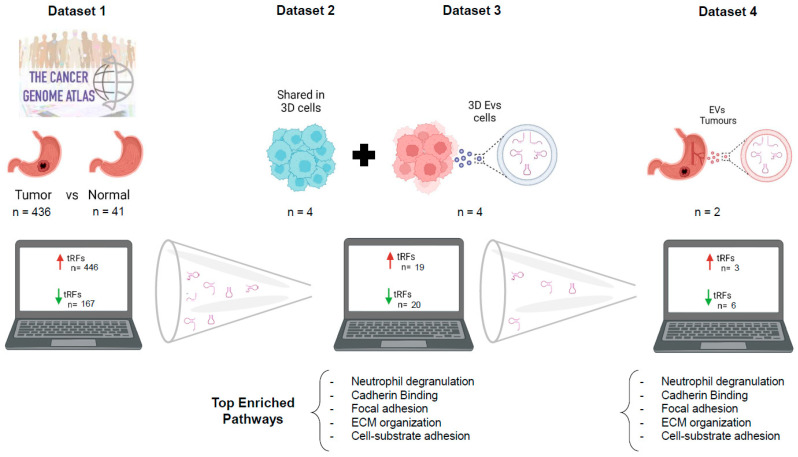
Data Summary. The strategy used to repurpose NGS data from public repositories and from previous in-house experiments allowed the identification of nine DE-tRFs present across all datasets that can have biomarker value in GC patients. Legend: red arrows: upregulated tRFs; green arrows: downregulated tRFs.

This work repurposing public and previously obtained data in our lab allowed the identification of a set of tRFs conserved across GC datasets that may work as GC diagnosis biomarkers (Figure 6). Remarkably, nine tRFs alone were representative of the largest tRF set correlating tumors, 3D-GC cells and patient-derived EV data, and were shown to target the same gene set responsible for TME modulation. Although this work needs experimental data validation regarding the effect of these vesicles in TME, the fact that this is predicted in four independent datasets strengthens the validity of this analysis. Future studies following similar workflows and validating results obtained with repurposed data can represent a cost-effective approach to novel research questions.

## 4. Materials and Methods

### 4.1. tRF Sequencing Data Collection and Pre-Processing

#### 4.1.1. TCGA

miRNA-aligned reads (bam files) released by TCGA were obtained from the GDC data portal (https://portal.gdc.cancer.gov/ (accessed on 13 November 2020)), filtering by TCGA–STAD project name. In total, 491 bam files were downloaded and converted to sequencing reads (fastq files) using biobambam2 [52]. Illumina Small RNA v1.5 adapter sequences were checked and clipped from all reads using cutadapt (version 2.8, with settings of “-a ATCTCGTATGCCGTCTTCTGCTTGT-q 20-m 16”) [53]. Low quality bases were trimmed from the 3′ end of the reads. After this quality control, all sequence reads shorter than 16 bases were discarded.

#### 4.1.2. S. Rocha et al.

On the other hand, 16 fastq files were generated corresponding to the small RNA sequencing reads from gastric cancer cell lines and EVs derived from them (methods reported in [9]). Ion Torrent adapter sequences were checked from all reads using cutadapt (version 2.8, with settings of “-b ATCACCGACTGCCCATAGAGAGG-q 20-m 16” [53]. Low quality bases were trimmed from the 3′ end of the reads. After this quality control, all reads shorter than 16 bases were discarded.

#### 4.1.3. GC Patients

In addition, we used 2 plasma samples of EVs from gastric cancer patients before undergoing surgery.

##### Blood Sample Collection from Gastric Cancer (GC) Patients

The 4 patients with Gastric Cancer (GC) enrolled in this study were admitted at Instituto Português de Oncologia Francisco Gentil (Coimbra, Portugal). This study was approved by the hospital’s ethics committee, and written informed consent was obtained from all patients before sample collection.

Peripheral blood samples (5 mL) were collected between April 2015 and May 2017 in K2 EDTA plasma preparation tubes (BD Vacutainer^®^ PPT) and processed into plasma within 30 min following the manufacturer’s instructions. Plasma samples were stored at −80 °C until further processing.

##### EV Isolation and Characterization from Plasma of GC Patients

Thawed plasma samples (volume 1.5–3 mL) were diluted with 0.9% NaCl (pH 7.4) to a final volume of 15 mL and filtered through a 0.22 µm filter. Filtered supernatants were centrifuged in an SW32 rotor (Beckman Coulter, Fullerton, CA, USA) at 100,000× *g*, for 14 h at 4 °C, to pellet EVs. EV pellets were washed in 0.9% NaCl (pH 7.4), centrifuged at 100,000× *g* for 2 h at 4 °C and resuspended in a volume of 0.9% NaCl. EVs were stored at 4 °C. EV size and concentration were further determined by Nanoparticle Tracking Analysis (NTA) using the NanoSight NS300 instrument (Malvern, Worcestershire, UK) with the scientific CMOS sensor. Briefly, EVs were diluted (1:500) in 0.9% NaCl. Three technical measurements were recorded under a controlled fluid flow with a pump speed set to 40 and a camera focus level adjusted between 10 and 16. The three videos were further analyzed using the NTA 3.1 Build 3.1.54 software to calculate the concentration, mode and mean size of EVs.

##### RNA Extraction from Human Plasma EVs (GC)

Prior to RNA isolation, EVs were incubated with RNAse A at 37 °C for 10 min (final concentration 0.4 mg/mL; NZYTech, Lisbon, Portugal). RNAse A was inhibited with the RNasin ribonuclease inhibitor (final concentration 1 U/μL; Promega, Madison, WI, USA). Next, EVs were treated with proteinase K at 37 °C for 10 min (final concentration 0.05 mg/mL; Qiagen, Hilden, Germany), which was inactivated at 75 °C for 10 min. Small RNA was isolated from RNAse A/proteinase K-treated EVs with the mirCURY RNA isolation kit—Biofluids (Exiqon, Vedbaek, Denmark), according to the manufacturer’s instructions. The concentration and quality of sRNA, including miRNA, were measured using the Agilent 2100 Bioanalyzer with the small RNA kit (Agilent, Santa Clara, CA, USA). Purified EV-RNA was kept at −80 °C until the further analysis.

##### Small RNA Library Preparation and Sequencing from Human Plasma EV-sRNA (GC)

Isolated sRNA was used for the small RNA library preparation using the Ion Total RNA-Seq Kit v2 (Thermo Fisher Scientific, Waltham, MA, USA), according to the manufacturer’s instructions. Briefly, 3′ and 5′ adapters were attached directionally, and simultaneously, to 3 μL of sRNA input (0.94–5.23 ng). Hybridized and ligated RNA was reversed transcribed using the Ion RT primer v2 and SuperScript III Enzyme mix. Each cDNA sample was amplified and barcoded using Platinum PCR SuperMix High Fidelity, the Ion Xpress RNA 3′ Barcode Primer, and a unique Ion Xpress RNA-Seq Barcode BC Primer, which allows sample identification and tracking. The size distribution of amplified cDNA was measured using the Agilent 2100 Bioanalyzer with the Agilent DNA 1000 Kit (Agilent, Santa Clara, CA, USA). Due to the high amount of adapter dimers, an adapted protocol was implemented with the aim of reducing the quantity of adapter dimers and enriching the library of interest. Library selection and a sequencing test run were performed. The first approach consisted of size selection with E-Gel^®^ SizeSelect™ 2% Agarose Gel (Thermo Fisher Scientific), which decreased the proportion of adapter dimers. For less concentrated libraries, size selection of the library was performed by using 4% Agarose Gel; cutting and purification of the band corresponding to ≈110 bp sequences was performed with illustra GFX PCR DNA and the Gel Band Purification Kit (GE Healthcare, New York, NY, USA). Agilent 2200 TapeStation (S/N 3-PM-1173NA)—HS D1000 Screen Tape (P/N 5067-5584) was used for size distribution control. An equal volume (3 µL) of each library was used to prepare the final pool. Pooled libraries were processed on Ion Chef System (S/N CHEF00657) using the Ion 540 Kit-Chef (P/N A27759) and the resulting 540 chip (P/N A27766) was sequenced on the Ion S5 XL System (S/N 245717100156). Fastq files were generated using the Torrent Suit plugin FileExporter v5.0.

##### Pre-Processing of Human Plasma EV-sRNA Sequencing Data (GC)

For the aim of this paper, we obtained 12 fastq files. FastQC (version 0.11.5) [54] was used to perform quality control checks on sequencing data. Remaining Ion Torrent sRNA adapter sequences were checked and clipped from all reads using cutadapt (version 2.8, with settings of “-b ATCACCGACTGCCCATAGAGAGGAAAGCGG—error-rate 0.2-times 1-m 15-q 20”) [53]. Low quality bases (q < 20) were trimmed from the 3′ end of the reads. Finally, all sequence reads shorter than 15 bases were removed.

### 4.2. tRF Expression Estimation

The processed reads were aligned to the human reference genome by MINTmap (default genome assembly GRCh37), specifically designed for tRF analyses [55]. MINTmap considers in the alignment the complex representation of the tRNA-derived sequences in the genome (i.e., regions shared between tRNA isodecoders, sequences that resemble mitochondrially encoded tRNAs in the nuclear genome, etc.) and, consequently, maps reads more exactly (no mismatches, insertions or deletions are allowed), deterministically (read matches are not based on probabilistic approaches), exhaustively (enumerates all possible alignments in the genome) and specifically (labels a tRF sequence as exclusive or ambiguous depending, respectively, on whether the tRF only belongs to a tRNA gene/s or it is also present in other positions in the genome (i.e., partial tRNA sequences or non-tRNAs), thus informing about the possibility that the tRF may be a false positive [56]. Only exclusive tRFs were considered in this study. The alignment of tRFs with MINTmap included a classification of tRFs according to five structural types (5′-tRFs, i-tRFs, 3′-tRFs, 5′-tRNA halves (5′-tRHs) and 3′-tRNA halves (3′-tRHs)) [55].

#### 4.2.1. TCGA

tRF expression levels of 446 gastric primary tumor samples and 41 NAT were calculated by MINTmap [55]. In total, 10 replicates from 446 primary tumors were removed, leaving those ones with a higher coverage, reaching a total of 436 tumors and 41 normal samples. Expression levels were tested for significant differential expression between the libraries of tumors and normal samples using the edgeR package (version 3.32.0) [57] in R (version 4.0.3) [58]. Data were normalized using the TMM method (weighted trimmed mean of M-values) [59]. Expression data from selected genes were used for heatmap construction depicting log_2_(cpm) values scaled to the z-score with supervised hierarchical clustering for dendrogram construction applying calculated Euclidean distances by R package “gplots” [60]. Venn diagrams were constructed by the jvenn tool [61].

A generalized linear model was fit after estimating the common, trended and tagwise dispersion. We noticed 22 groups of samples related to the center where the samples were sequenced; therefore, we included cancer status and batch as fixed effects in the model. The likelihood ratio test was used to evaluate differential expression between allergic and non-allergic donors. Differences in expression between conditions with |log_2_(fold-change)| > 1 and an adjusted *p*-value of <0.05 were considered to be significant. *p*-values were adjusted for the false discovery rate with the Benjamini–Hochberg method [62].

#### 4.2.2. GC Study

tRF expression levels of 4 gastric cancer cell and 4 EV samples were calculated by MINTmap [55]. For cells, we considered those tRFs with >10 reads globally, and for EVs, those with >1 read were considered.

#### 4.2.3. GC Patient EVs

tRF expression levels of 12 gastric cancer EV samples from blood were calculated by MINTmap [55]. We considered those tRFs with >1 read in samples collected before surgery from 2 patients, which had an acceptable sequencing quality.

### 4.3. Target Prediction

We used the tRFTar [63] database to predict the targets of the tRF of interest. The *ClusterProfiler* R package was used for the assessment of significantly enriched GO terms and pathways (*padj* < 5.00 × 10^−2^). tRF–target gene interaction (TGI) networks were created by the R package igraph (version 1.2.6) [64]. Cytoscape (version 3.8.0) [65] was used to depict the networks.

## Figures and Tables

**Table 1:** Table showing relevant information about the top 15 DE-tRFs in GC tumors when compared to NAT in the TCGA dataset (ranked by *p*-value). Legend: UNIQUE—tRF derived from unique tRNA isodecoder; MT—tRF derived from a mitochondrial tRNA; AMBIGUOUS—tRF may be derived from >1 tRNA isodecoder.

Name	tRF_Type	Sequence	Exclusivity	Anticodon	logFC	logCPM	LR	Pvalue	FDR
tRF-23-YJE76INB0J	i-tRF	TTAGCACTCTGGACTCTGAATCC	UNIQUE	GlnCTG	−3.47878163	10.6598912	118.981005	1.06 × 10^−27^	1.04 × 10^−24^
tRF-22-91PJB7MNK	i-tRF	TGGCCGCAGCAACCTCGGTTCG	UNIQUE	HisGTG	−4.01790481	9.10726781	118.346426	1.46 × 10^−27^	1.04 × 10^−24^
tRF-22-WB8US5652	3′-tRF	TCGAATCCGAGTCACGGCACCA	UNIQUE	HisGTG	−2.19175055	8.94123967	116.35393	3.98 × 10^−27^	1.90 × 10^−24^
tRF-21-91PJB7MND	i-tRF	TGGCCGCAGCAACCTCGGTTC	UNIQUE	HisGTG	−3.54575799	7.36208523	100.300282	1.31 × 10^−23^	4.69 × 10^−21^
tRF-22-VF4YO9XEJ	i-tRF	TAGCACTCTGGACTCTGAATCC	UNIQUE	GlnCTG	−3.21016875	10.1858067	99.1946855	2.29 × 10^−23^	6.55 × 10^−21^
tRF-24-SWRYVMMVHX	i-tRF	GTCGTGGTTGTAGTCCGTGCGAGA	MT	GluTTC	6.01156036	10.0232838	93.2423782	4.63 × 10^−22^	1.10 × 10^−19^
tRF-21-WB8US565D	3′-tRF	TCGAATCCGAGTCACGGCACC	UNIQUE	HisGTG	−2.15755385	7.62862441	87.2918519	9.36 × 10^−21^	1.92 × 10^−18^
tRF-23-91PJB7MNDL	i-tRF	TGGCCGCAGCAACCTCGGTTCGA	UNIQUE	HisGTG	−4.27131139	7.79586266	82.6891628	9.60 × 10^−20^	1.72 × 10^−17^
tRF-23-VF4YO9XED2	i-tRF	TAGCACTCTGGACTCTGAATCCA	UNIQUE	GlnCTG	−2.77409913	9.43587704	77.8958834	1.09 × 10^−18^	1.73 × 10^−16^
tRF-21-EXEY0VWUD	3′-tRF	ACTTAACTTGACCGCTCTGAC	MT	ValTAC	3.47697974	12.9374387	76.9701494	1.74 × 10^−18^	2.49 × 10^−16^
tRF-24-8DYDZDL9JR	3′-tRF	TCAACTTAACTTGACCGCTCTGAC	MT	ValTAC	3.34575605	9.93002784	74.5263922	5.98 × 10^−18^	7.79 × 10^−16^
tRF-22-8B8SOUPR2	3′-tRF	TCAAATCCCGGACGAGCCCCCA	AMBIGUOUS	ProAGG	−1.86693881	9.09960168	73.4251504	1.05 × 10^−17^	1.25 × 10^−15^
tRF-20-NONU3IND	3′-tRF	CTTAACTTGACCGCTCTGAC	MT	ValTAC	3.10311975	10.6738238	73.1309279	1.21 × 10^−17^	1.34 × 10^−15^
tRF-18-INVDRID1	i-tRF	ATGTTTAGACGGGCTCAC	MT	PheGAA	−2.7987848	8.31865382	72.1257612	2.02 × 10^−17^	2.07 × 10^−15^
tRF-23-ZVELXKKSDZ	i-tRF	TTTGCACGTATGAGGCCCCGGGT	UNIQUE	AlaTGC	−2.02023747	6.92621223	70.3849902	4.88 × 10^−17^	4.66 × 10^−15^

**Table 2:** tRFs present across all datasets. Table showing relevant information about the nine DE-tRFs present in patient-derived EVs.

tRF_ID	tRF_Type	tRF_Sequence	Exclusive	Anticodon	Tumor vs. NAT (TCGA)
tRF-16-RPM830D	5′-tRF	GGTAGCGTGGCCGAGC	AMBIGUOUS	LeuAAG	Downregulated
tRF-17-8R1546J	3′-tRF	TCCCCAGTACCTCCACC	UNIQUE	AlaAGC	Upregulated
tRF-18-69M8LO04	5′-tRF	GGCTCCGTGGCGCAATGG	UNIQUE	ArgTCT	Upregulated
tRF-18-8R1546D2	3′-tRF	TCCCCAGTACCTCCACCA	UNIQUE	AlaAGC	Upregulated
tRF-18-YRRHQFD2	3′-tRF	TTCCCGGGCGGCGCACCA	UNIQUE	GlyCCC	Downregulated
tRF-22-8EKSP1852	3′-tRF	TCAATCCCCGGCACCTCCACCA	UNIQUE	AlaAGC	Downregulated
tRF-22-WD8S746D2	3′-tRF	TCGACTCCCGGTGTGGGAACCA	UNIQUE	GluTTC	Downregulated
tRF-22-WE8RO86J2	3′-tRF	TCGATTCCCCGACGGGGAGCCA	UNIQUE	AspGTC	Downregulated
tRF-22-WEKSPM852	3′-tRF	TCGATCCCCGGCATCTCCACCA	AMBIGUOUS	AlaTGC	Downregulated

## Data Availability

All data will be made available upon reasonable request to the corresponding author.

## Supplementary Materials

The supporting information can be downloaded at: https://www.mdpi.com/article/10.3390/ijms24108961/s1.
