# Supplementary material for: NGS Data Repurposing Allows Detection of tRNA Fragments as Gastric Cancer Biomarkers in Patient-Derived Extracellular Vesicles
Source: Int J Mol Sci. 2023 May 18;24(10):8961. doi: 10.3390/ijms24108961 (PMC10219402; doi:10.3390/ijms24108961)
Supplement: Supplementary file 1 [file ijms-24-08961-s001.zip › ijms-2313829-supplementary.pdf]

Supplementary Files Maqueda & Santos et al. 2023

MINTbase.Unique.ID	rank.394.expr.in.EVs	rank.394.expr.in.cells
**tRF-18-R29P4P04**	14	88
**tRF-30-P4R8YP9LON4V**	30	25
**tRF-20-Q99P9P9N**	34	267
**tRF-18-69M8LO04**	44	28
**tRF-18-8R1546D2**	45	5
**tRF-29-FP18LPMBQ4H4**	54	51
**tRF-19-69M8LOJX**	56	45
**tRF-19-S998LOJ4**	57	40
**tRF-28-P4R8YP9LOND5**	73	33
**tRF-18-O7M8LO04**	100	92
**tRF-29-86J8WPMN1EJ3**	145	254
**tRF-17-8R1546J**	152	99
**tRF-19-RKIP4OF4**	164	324
**tRF-29-P4R8YP9LONHK**	172	86
**tRF-18-O7M8LOD4**	187	252
**tRF-23-R29P4P9LDS**	197	132
**tRF-18-Q99P9P04**	224	323
**tRF-29-PSQP4PW3FJFL**	252	257
**tRF-19-R9JP9P1Z**	313	246

Supplementary Table S1: tRF rank expression in 3D-cells and derived EVs. There is no correlation between the expression rank in 3D-cells and EVs, suggesting that tRF loading into EVs is not random.

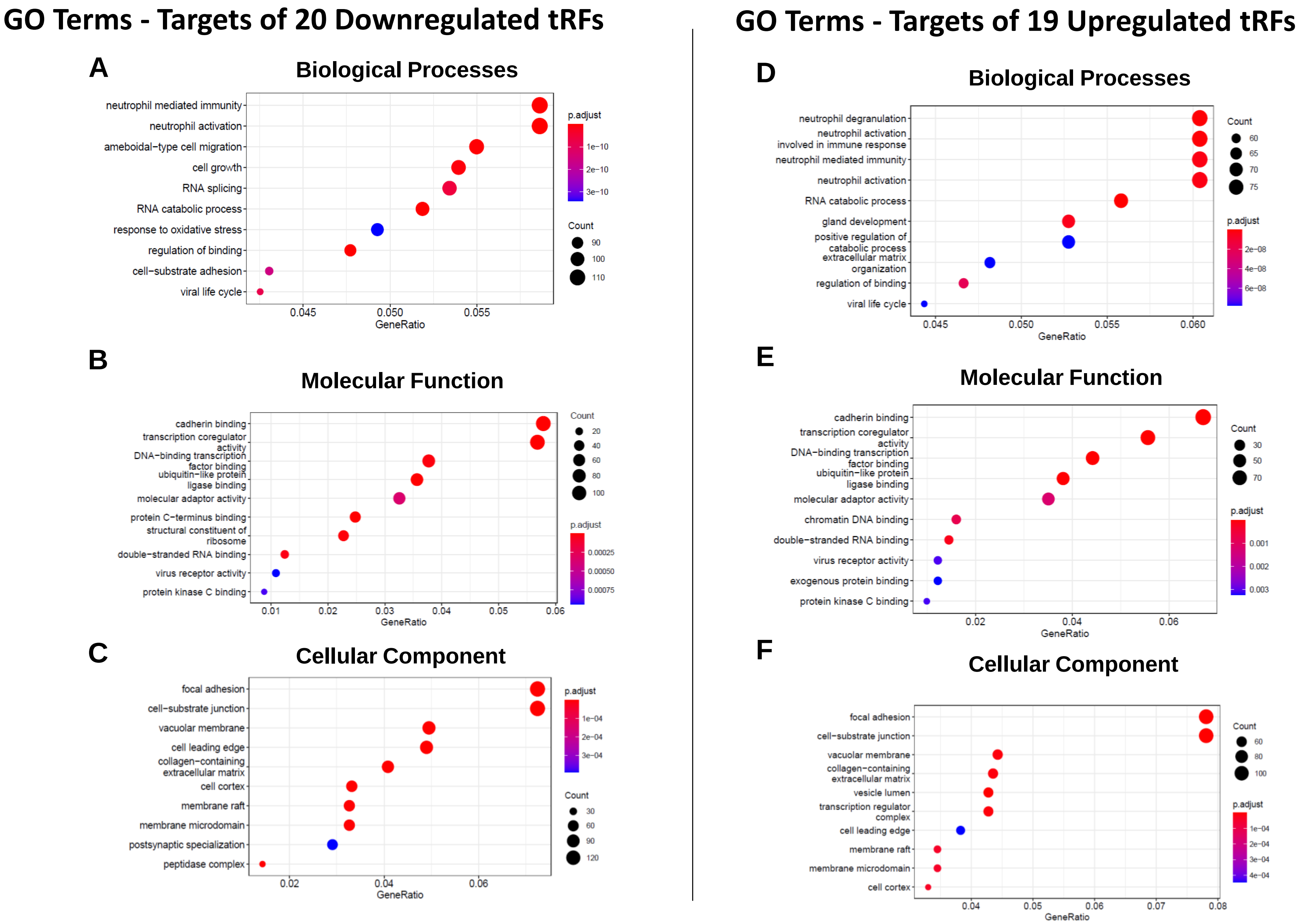

Supplementary Figure S1: Up or downregulated tRFs in TCGA vs EVs that are also present in *in vitro* datasets target genes with similar functions. Dotplots showing GO-terms group in Biological Processes, Molecular Function and Cellular Component, predicted to be impacted by the 20 downregulated tRFs (A-C) and the 19 upregulated tRFs (D-F).
